# Event Analysis: Using Transcript Events To Improve Estimates of Abundance in RNA-seq Data

**DOI:** 10.1534/g3.118.200373

**Published:** 2018-07-18

**Authors:** Jeremy R. B. Newman, Patrick Concannon, Manuel Tardaguila, Ana Conesa, Lauren M. McIntyre

**Affiliations:** * Department of Molecular Genetics and Microbiology and Genetics Institute, University of Florida, Gainesville, Florida; † Genetics Institute and Department of Pathology, Immunology and Laboratory Medicine, University of Florida, Gainesville, Florida; ‡ Department of Microbiology and Cell Science, Institute for Food and Agricultural Sciences, University of Florida, Gainesville, Florida, 32610; Wellcome Trust Sanger Institute, Hinxton, United Kingdom; § Department of Microbiology and Cell Science, Institute for Food and Agricultural Sciences; Genetics Institute, University of Florida, Gainesville, Florida, 32610; Genomics of Gene Expression Lab, Prince Felipe Research Center, Valencia, Spain; ** Department of Molecular Genetics and Microbiology and Genetics Institute, University of Florida, Gainesville, Florida

**Keywords:** RNA sequencing, gene expression, transcriptomics, alternative splicing, isoform estimation

## Abstract

Alternative splicing leverages genomic content by allowing the synthesis of multiple transcripts and, by implication, protein isoforms, from a single gene. However, estimating the abundance of transcripts produced in a given tissue from short sequencing reads is difficult and can result in both the construction of transcripts that do not exist, and the failure to identify true transcripts. An alternative approach is to catalog the events that make up isoforms (splice junctions and exons). We present here the Event Analysis (EA) approach, where we project transcripts onto the genome and identify overlapping/unique regions and junctions. In addition, all possible logical junctions are assembled into a catalog. Transcripts are filtered before quantitation based on simple measures: the proportion of the events detected, and the coverage. We find that mapping to a junction catalog is more efficient at detecting novel junctions than mapping in a splice aware manner. We identify 99.8% of true transcripts while iReckon identifies 82% of the true transcripts and creates more transcripts not included in the simulation than were initially used in the simulation. Using PacBio Iso-seq data from a mouse neural progenitor cell model, EA detects 60% of the novel junctions that are combinations of existing exons while only 43% are detected by STAR. EA further detects ∼5,000 annotated junctions missed by STAR. Filtering transcripts based on the proportion of the transcript detected and the number of reads on average supporting that transcript captures 95% of the PacBio transcriptome. Filtering the reference transcriptome before quantitation, results in is a more stable estimate of isoform abundance, with improved correlation between replicates. This was particularly evident when EA is applied to an RNA-seq study of type 1 diabetes (T1D), where the coefficient of variation among subjects (n = 81) in the transcript abundance estimates was substantially reduced compared to the estimation using the full reference. EA focuses on individual transcriptional events. These events can be quantitate and analyzed directly or used to identify the probable set of expressed transcripts. Simple rules based on detected events and coverage used in filtering result in a dramatic improvement in isoform estimation without the use of ancillary data (*e.g.*, ChIP, long reads) that may not be available for many studies.

## Background

RNA-seq is a powerful tool for profiling gene expression, and has been employed to quantitate expression, and infer allele specific expression and alternative splicing (*e.g.*, (Mortazavi *et al.* 2008; Main *et al.* 2009; Wang *et al.* 2009; Montgomery *et al.* 2010; Nagalakshmi *et al.* 2010; Pastinen 2010; Graze *et al.* 2012; Dalton *et al.* 2013; Korir and Seoighe 2014; Leon-Novelo *et al.* 2014; Akin *et al.* 2016; Fear *et al.* 2016; Goldstein *et al.* 2016; Kang *et al.* 2016; Nellore *et al.* 2016; Newell *et al.* 2016)). The importance of alternative splicing has led to the development of numerous algorithms to estimate isoform abundance from RNA-seq data, including Cufflinks (Trapnell *et al.* 2012), RSEM (Li *et al.* 2010; Li and Dewey 2011), and eXpress (Roberts *et al.* 2011; Roberts and Pachter 2013), and more recently iReckon (Mezlini *et al.* 2013) and CIDANE (Canzar *et al.* 2016), and others (*e.g.*, (Jiang and Wong 2009; Nicolae *et al.* 2011; Turro *et al.* 2011; Li and Jiang 2012; Sun 2012; Sturgill *et al.* 2013; Glaus *et al.* 2012; Patro *et al.* 2014; Nariai *et al.* 2014; Lee *et al.* 2015)). The accurate identification of an individual transcript requires the presence of at least one exon or splicing event unique to that transcript (Cloonan *et al.* 2008; Liu *et al.* 2016b). However, there are transcript isoforms that contain no events unique to that isoform. Even when a unique event is detected in one isoform, reads mapping to non-unique portions of the transcript cannot be assigned with certainty. Recent evaluations conclude that while some algorithms, such as RSEM, perform better than others in simulations or particular example data, there are, unsurprisingly, errors in all current methods (Angelini *et al.* 2014; Kanitz *et al.* 2015; Ding *et al.* 2017; Williams *et al.* 2017; Tardaguila *et al.* 2018). Alternatives to isoform estimation include focusing on differential abundance of junctions (Zhang *et al.* 2012; Rezaeian *et al.* 2016) and alternative exon inclusion (Katz *et al.* 2010). These event-based approaches have the benefit of not propagating the uncertainty of an isoform estimate in inferences about splicing. Tests of differential splicing are then exon based or exon/junction based (*e.g.*, (Anders *et al.* 2012)). However, there are drawbacks with these approaches, as currently implemented, that include an increased multiple testing burden, difficulties in making inferences about the impact of splicing for a particular gene, and challenges in identifying patterns in results.

Here, we generalize the event- or feature-based analysis approach to assessing alternative splicing, resulting in a number of improvements in sensitivity and specificity, and in improved replicate-to-replicate concordance of transcript estimates. We take advantage of prior observations from long read PacBio data indicating that, while there are many novel isoforms detected (Sharon *et al.* 2013; Tombácz *et al.* 2016; Wang *et al.* 2016; Tardaguila *et al.* 2018), most are new combinations of known components (Au *et al.* 2013; Tardaguila *et al.* 2018). Nellore *et al.* (Nellore *et al.* 2016) studied more than 20,000 human RNA-seq samples derived from multiple cell types and found that that only 3.5% of junctions are not derivable in from existing genome annotations in some form, and that 81.4% are from already annotated transcripts. We also note that in the literature, there are examples of identified novel transcripts of several genes that are comprised of new combinations of known splice sites, frequently those that are the result of a variant inducing an exon-skipping event (Booms *et al.* 1999; Hide *et al.* 2001; Zhou *et al.* 2010; Lim *et al.* 2011; Eswaran *et al.* 2013; Kim *et al.* 2013b; Gabreski *et al.* 2016). This suggests that an augmented reference consisting of probable new events based on the set of existing transcripts can be developed. Here we present Event Analysis (EA), a strategy for pre-filtering sample-specific transcriptomes to improve transcript quantification. Basically, we expand the reference catalog by including all plausible events based on known exons and annotate all events. Then, for a particular RNA-seq sample, we quantitate events and use this information to calculate summary measures for transcripts which are used to filter the transcriptome of that sample. When the resulting filtered set of likely expressed transcripts is used as input for transcript quantification by RSEM (Li and Dewey 2011) or eXpress (Roberts and Pachter 2013) the correlation between replicates is improved compared to use of the unfiltered reference. Mapping to an expanded catalog identifies more true junctions compared to mapping using splice aware techniques (Dobin *et al.* 2013) both in simulation and in data from mouse neural progenitor cells. Filtering based on events results in more accurate estimates of the transcriptome than a state of the art combination *de novo/*annotation method iReckon (Mezlini *et al.* 2013). In a large scale RNA-seq experiment in different cell types of ∼100 individuals with type 1 diabetes (T1D) we show that our approach substantially decreases the coefficient of variation for estimates of isoform abundance across samples.

## Methods

### Simulated data

Polyester (Frazee *et al.* 2015) was used to simulate reads for the mouse genome based on two scenarios. For the first simulation (Simulation 1), a set of highly expressed genes with few isoforms per gene was designed. This represents a best case scenario. Here, 10,000 RefSeq transcripts were randomly selected. The only restriction that was placed on transcript selection was that it could not come from a gene with an exonic sequence shared across more than one gene, thus avoiding regions of genic ambiguity. The results was a set of 10,000 transcripts representing 7,876 genes. Paired end reads were simulated for 6 replicates at 100 × coverage (approximately 53.4 million paired end reads per sample). Read size was set to 2×56 bp (matching the mouse neural data below). A second simulation (Simulation 2) was performed using all 467 annotated RefSeq transcripts from 59 genes, and represents a small-scale scenario where all transcripts of a gene are expressed (approximately 1.4 million paired end reads per sample). Genes were selected to represent a variety of difficult to resolve multi-transcript situations – the worst case scenario (see File S1). These simulated datasets are available at https://github.com/McIntyre-Lab/events.

### Mouse RNA-seq data

RNA-seq data from mouse neural progenitor cells (NPCs) and oligodendrocyte precursor cells (OPCs) used in this analysis were generated as described in Tardaguila *et al.* (Tardaguila *et al.* 2018) and are available from the NCBI Sequence Read Archive (study accession number SRP101446). Briefly, NPCs were isolated from the subventricular zone of killed neonatal c57/BL6 mice. NPCs were cultured as neurospheres in EGF/bFGF-supplemented media, and OPCs were derived by differentiating NPCs with All Trans Retinoic Acid. Total RNA was isolated using the Nucleospin RNA kit (Macherey-Nagel). Two biological replicates were performed for each cell type. RINs (RNA Integrity Numbers) were between 10 and 9.7 for all samples. Full-length cDNA was synthesized using the SMARTer PCR cDNA Synthesis kit (Clontech, version 040114) following PacBio recommendations. The reaction input was 1 µg of total RNA. Two first-strand cDNA synthesis reactions were performed per sample. First strand cDNA was then divided into nine PCR reactions ultimately yielding approximately 14–16 µg full-length cDNA per sample (Tardaguila *et al.* 2018). The same cDNA preparation from each sample was used to prepare both Illumina and PacBio sequencing libraries. Illumina sequencing was performed at the Interdisciplinary Center for Biotechnology Research (University of Florida) with the Illumina Nextseq instrument using Nextera tagmentation, resulting in approximately 60 million 56 bp single end reads per sample (Tardaguila *et al.* 2018).

### Mouse long read validation data

PacBio sequencing was performed as per the Iso-Seq protocol using P4-C2 chemistry at the Interdisciplinary Center for Biotechnology Research (University of Florida). The same cDNA used for the RNA-seq experiments was fractionated into three fractions (1-2kb, 2-3kb and 3-6kb) using BluePippin and sequenced on the RSII instrument using a total of eight SMRT cells per sample (two SMRT cells for the 1-2kb fraction, three SMRT cells for the 2-3kb and 3-6kb fractions), generating approximately 0.6M reads of insert (Tardaguila *et al.* 2018).

### T1D RNA-seq data

RNA-seq data were obtained from three major classes of lymphocyte (CD4+ T cells, CD8+ T cells and CD19+ B cells) from 82 T1D cases from the Type 1 Diabetes Genetics Consortium, as previously described (Newman *et al.* 2017), and is available from NCBI database of Genotype of Phenotypes (dbGaP), accession number phs001426.v1.p1. Briefly, peripheral blood mononuclear cells were fractionated by positive selection on antibody-coated magnetic beads into the three lymphocyte populations (CD4^+^ T cells, CD8^+^ T cells, CD19^+^ B cells). Purities of these populations (>90%) were confirmed by flow cytometry. RNA was purified and libraries were prepared and sequenced (approximately 50 million 2×50bp reads per sample) in three pools using the Illumina HiSeq 2000 platform at the HudsonAlpha Genome Services Laboratory (Huntsville, Alabama). Individual cell samples with low coverage were excluded from all analyses (2 CD4+ samples, 2 CD8+ samples, 1 CD19+ sample). In total, there were 81 subjects with sequencing data from at least one cell type were included, of which 79 subjects had usable sequencing data from all three cell types (Newman *et al.* 2017).

### Event Analysis

#### Creation of the Junction catalog:

**Figure 1:**
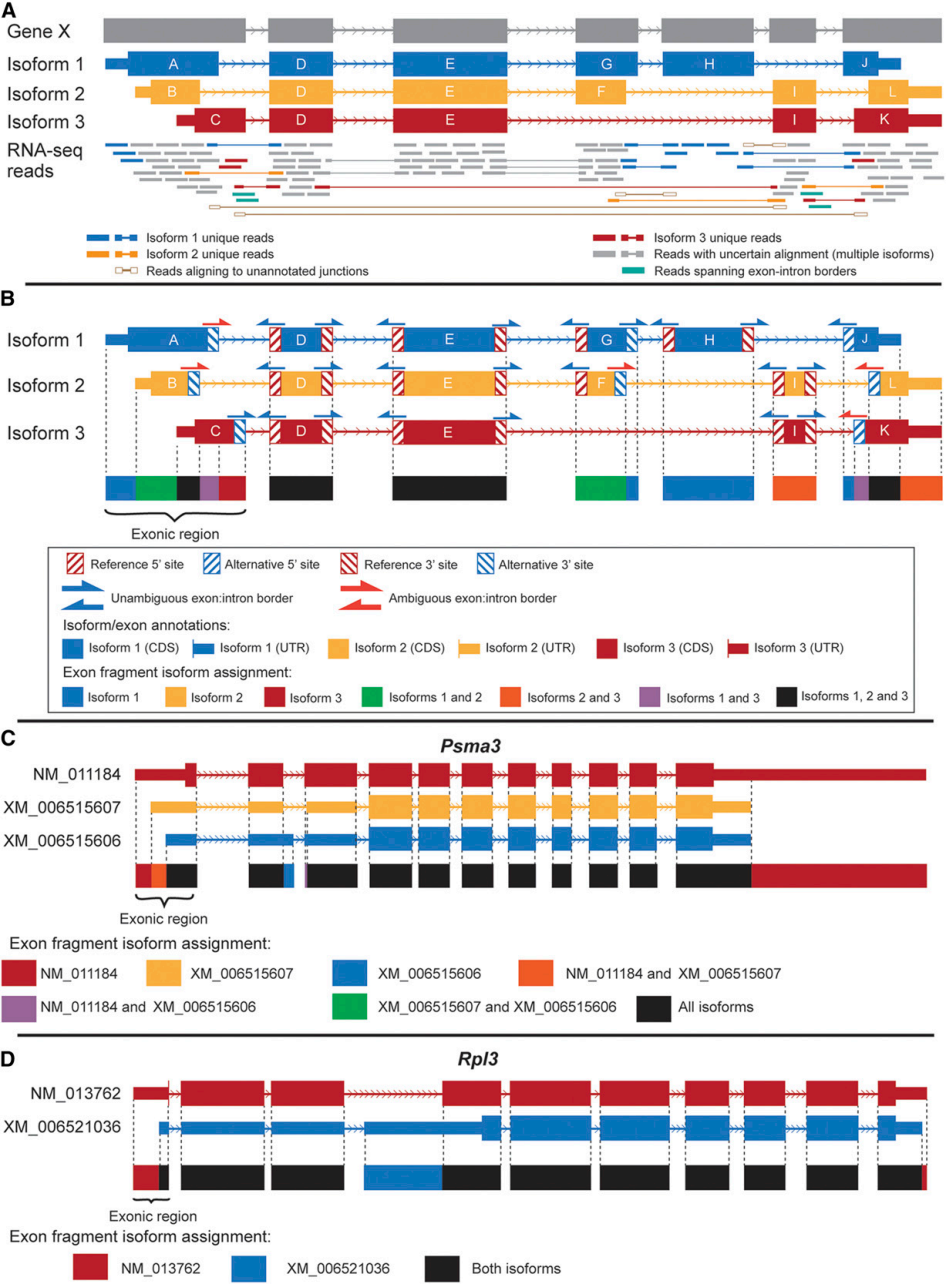
(A) Example of alignment of reads to three transcripts of an example gene, and are displayed relative to their positions in the gene. Exons are labeled sequentially by their 5′ start and 3′ end positions relative to the orientation of their respective gene, and exons with the same 5′ start and 3′ end positions are considered to be the same exon. Reads mapping to exonic regions or exon-intron borders are indicated by single bars, and reads mapping to junctions are denoted by pairs of bars connected by a line. Reads can map to sequences unique to a single transcript (blue, yellow, red), multiple transcripts (gray), theoretical junctions not present in any transcript (brown with white fill) or exon-intron boundaries (teal). (B) Exons, exonic regions and exon fragments. This example shows exons that differ in donor and acceptor sites. The exonic region is defined as the genome co-ordinates where overlapping exons from different transcripts are found. Within the exonic region, sequences can be classified based upon the exons that are annotated in the region. These exon fragments may be shared across all exons (black), unique to one exon (blue, yellow, read) or common to a subset of exons (orange, green, purple). Within an exonic region, 3′ (donor) sites and 5′ (acceptor) sites are classified as either the reference site (blue hatching) or an alternative site (red hatching). For each exonic region, a single border junction is selected from sequences corresponding to unambiguous exon-intron borders. The exonic regions that comprise of only a single exon (*i.e.*, exons D, E, H and I) are termed single-exons. By definition single-exons have no fragments, and can be unique (exon G), common (exon H) or constitutive (exon D and E) depending on their isoform membership. Examples of exons, exonic regions and exon fragments are demonstrated with two real genes from the mm10 RefSeq annotations: (C) *Psma3* (Entrez ID: 19167), consisting of 11 exonic regions and three transcripts that share most of the same events; and (D) *Rpl3* (Entrez ID: 27367), consisting of 10 exonic regions and two transcripts.

The first step of the EA approach creates a junction catalog starting with existing junctions, and augment this catalog by creating all possible logical junctions based on the set of known exons and map reads directly to junctions. Starting with a GFF3 file (*e.g.*, Ensembl, RefSeq, UCSC, AceView, or an individually curated custom reference) all junctions from logical combinations of exon pairs within the gene going from 5′-to-3′ were generated (Figure 1). By extending the 3′ sequence of a donor exon, or the 5′ region of an acceptor exon into the neighboring intron, putative novel exon donor/acceptors can be identified (Figure 1A, teal reads; Figure 1B, blue arrows above exons; Table 1; Table 2; Table S1, Additional File 1). To ensure that only currently annotated intronic sequences are evaluated for putative novel donor/acceptor sites, the junction from the 5′-most donor exon and the junction from 3′-most acceptor exon were used as the starting points. The complete catalog contains all previously annotated junctions; unannotated junctions that are novel combinations of existing donor/acceptor sites; and potential novel new donor/acceptor sites. The detection of previously unannotated junctions provides evidence that a gene may be producing a novel isoform (*e.g.*, see Figure 1A, brown unfilled reads). We adopt the naming convention used by AStalavista (Foissac and Sammeth 2007) and others, where each junction is identified by the combination of chromosome (or scaffold, contig, etc.), the last position of the donor exon, first position of the acceptor exon, and strand (*i.e.*, [chromosome]:[donor position]:[acceptor position]:[strand]).

**Table 1:** All splice junctions derived from transcripts the gene model in Figure 1 example

Splice junction *^a^*	Isoform	Annotated? (Y/N)	Annotated frequency (unique, common, constitutive)	Exon-skipping? (Y/N)	Alternative donor? (Y/N)	Alternative acceptor? (Y/N)
Exon A:Exon D	Isoform 1	Y	Unique	N	Y	N
Exon B:Exon D	Isoform 2	Y	Unique	N	Y	N
Exon C:Exon D	Isoform 3	Y	Unique	N	Y	N
Exon D:Exon E	Isoforms 1, 2 and 3	Y	Constitutive	N	N	N
Exon E:Exon F|Exon E:Exon G*	Isoform 1 (E:G), Isoform 2 (E:F)	Y	Common	N	N	N
Exon E:Exon I	Isoform 3	Y	Unique	Y	N	N
Exon F:Exon I	Isoform 2	Y	Unique	Y	Y	N
Exon G:Exon H	Isoform 1	Y	Unique	N	Y	N
Exon H:Exon J	Isoform 1	Y	Unique	Y	N	Y
Exon I:Exon K	Isoform 3	Y	Unique	N	N	Y
Exon I:Exon L	Isoform 2	Y	Unique	N	N	Y

a Junctions here are denoted with the 5′most exon first. The second exon/intron is separated from the first using a colon. Events with identical donors and acceptors are separated by a “|”

* Junction is not distinct as exons F and G share the same donor site.

**Table 2:** Examples of other possible, logical splice junctions derived from the gene model in Figure 1 example

Splice junction *^a^*	Annotated? (Y/N)	Annotated frequency (unique, common, constitutive)	Exon-skipping? (Y/N)	Alternative donor? (Y/N)	Alternative acceptor? (Y/N)	Exon-intron border? (Y/N)
Exon A:Exon F|Exon A:Exon G*	N	n/a	Y	Y	N	N
Exon A:Exon E	N	n/a	Y	Y	N	N
Exon C:Exon H	N	n/a	Y	Y	N	N
Exon A:Exon I	N	n/a	Y	Y	N	N
Exon B:Exon J	N	n/a	Y	Y	Y	N
Exon D:Exon J	N	n/a	Y	N	Y	N
Exon D:Exon K	N	n/a	Y	N	Y	N
Exon H:Exon K	N	n/a	Y	N	Y	N
Exon H:Exon L	N	n/a	Y	N	Y	N
Exon I:Exon J	N	n/a	N	N	Y	N
Exon C donor:intron	N	n/a	N	Y	N	Y
Exon E donor:intron	N	n/a	N	N	N	Y
Exon H acceptor:intron	N	n/a	N	N	N	Y

Full list of all possible junctions are listed in Supplementary Table 1 in Additional File 1.

a Junctions here are denoted with the 5′most exon/intron first. The second exon/intron is separated from the first using a colon. Events with identical donors and acceptors are separated by a “|”

* Junction is not unique as exons F and G share the same acceptor site.

ES = exon skipping, AD = alternative donor, AA = alternative acceptor.

#### Exonic regions:

The second step of event analysis is to identify all the exonic regions. In higher eukaryotes, exons may overlap with one another (*e.g.*, due to alternative donors, alternative acceptors, alternative transcription initiation/termination sites, etc.). The exonic region may be quantified without regard to differences in donor/acceptor sites (Dalton *et al.* 2013; Graze *et al.* 2014; Fear *et al.* 2016; Newell *et al.* 2016) to avoid double counting reads. In regions where differences are small (less than 10 bp) there is no meaningful loss of information in this approach. However, where exonic regions are comprised of overlapping exons that differ measurably, the 5′ and 3′ positions of exons within the exonic region are used to separate the region into exon fragments. EA annotates each exon fragment within an exonic region to indicate whether it is exclusive to a single exon or is shared among sets of exons (Figure 1B) and is further annotated to transcripts.

#### Annotating events:

**Figure 2:**
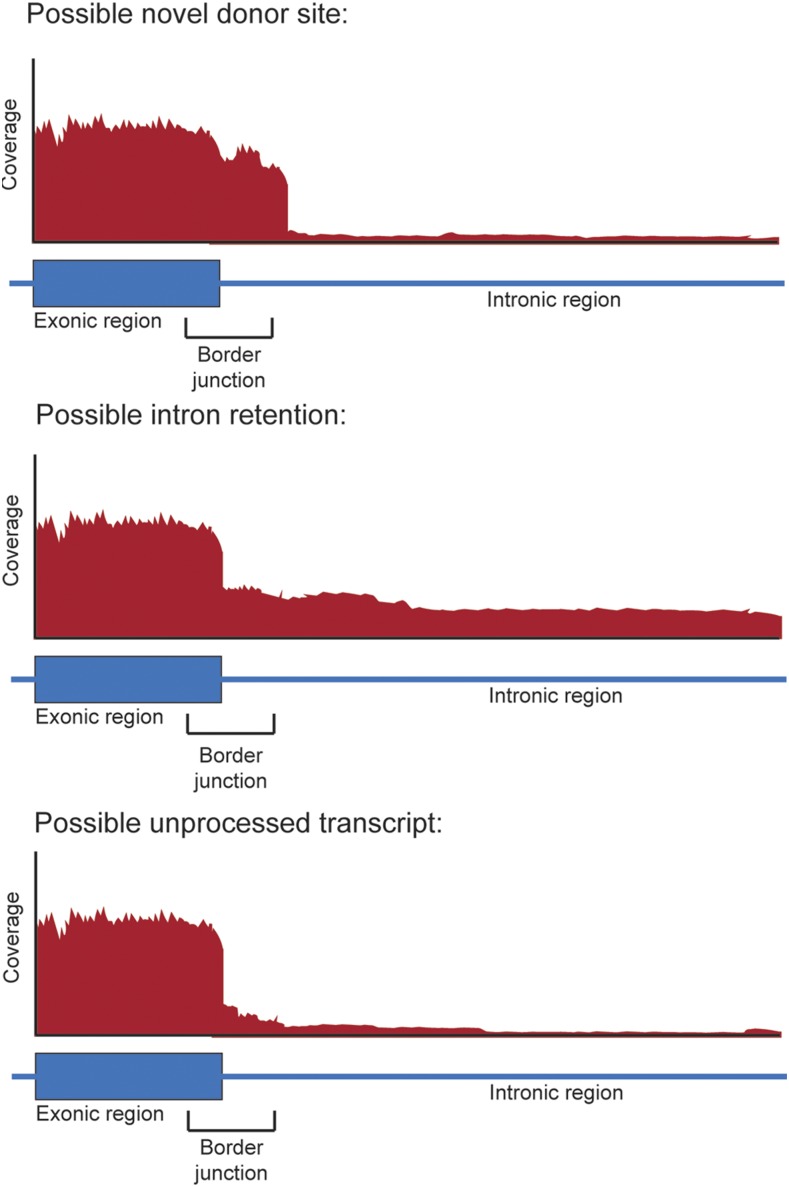
Possible classifications for border junctions. Detected border junctions are only classified if their adjacent exon is also detected. These border junctions can be categorized as a possible novel donor/acceptor, and possible intron retention. If the coverage at the border junction was similar in coverage to its adjacent exon (defined as the border junction having a mean APN of at least 90% of the APN of the exon), then the border junction is classified as a possible novel donor. If the coverage (as APN) of the border junction was at least 10% of the adjacent exon and the adjacent intron was also detected, then the border junction is classified as possible intron retention. These values are conservative and can be changed. Where a border junction could be classified as both a possible novel donor and possible intron retention, the event is classified as ambiguous intron retention. All remaining border junctions are classified as possible unprocessed transcript.

EA annotates junctions based on whether they exclude exons (exon-skipping splice junctions) or if they use alternative 5′ and/or 3′ splice sites (alternative donor junctions, alternative acceptor junctions). Exon-skipping junctions are defined as splice junctions that exclude (“skip”) one or more exons that are situated in the reference annotation between the donor and acceptor site of the junction (Figure 1, *e.g.*, junction between exons E and I; Table 1). Where there are multiple possible donor and/or acceptor sites within a group of overlapping exons, all donors/acceptors are classified as “alternative”. This follows a similar convention to other splicing definitions, such as those used by AStalavista (Foissac and Sammeth 2007). Junction definitions are not mutually exclusive: a junction can utilize both alternative donor and alternative acceptor sites and also exclude one or more intermediary exons (see examples in Figure 1, Table 1). The unannotated, putative novel donor/novel acceptor sites may reflect unprocessed transcript and intron retention events. To accommodate this uncertainty EA annotates these as border junctions. The likelihood of truly novel donor/acceptor sites is evaluated in a separate step based on the experimental data that includes mapping information from the adjoining exons/intron region. (Figure 2).

Events – junctions, exonic regions, and exon fragments – that are only annotated in a single isoform in the reference database are classified as “unique”. Events that are common to a multiple but not all isoforms are classified as “common”, while events that are annotated in all isoforms are classified as “constitutive”. Events that are not annotated to any isoform are classified as “unannotated”. Non-unique sequences can also result from the same event being shared in a set of transcripts as well as genes within the same gene family that are highly homologous. Uniqueness is expected to increase with longer reads. When events are not unique among different genes, it is classified as “multi-gene”. The complexity of resolving multi-gene events is well known (Djebali *et al.* 2012; Treangen and Salzberg 2012) and multi-gene events are not analyzed further. As more experiments are strand specific, the incidence of multi-gene junctions and multi-gene exonic regions will be reduced.

#### Mapping to the junction catalog:

**Figure 3:**
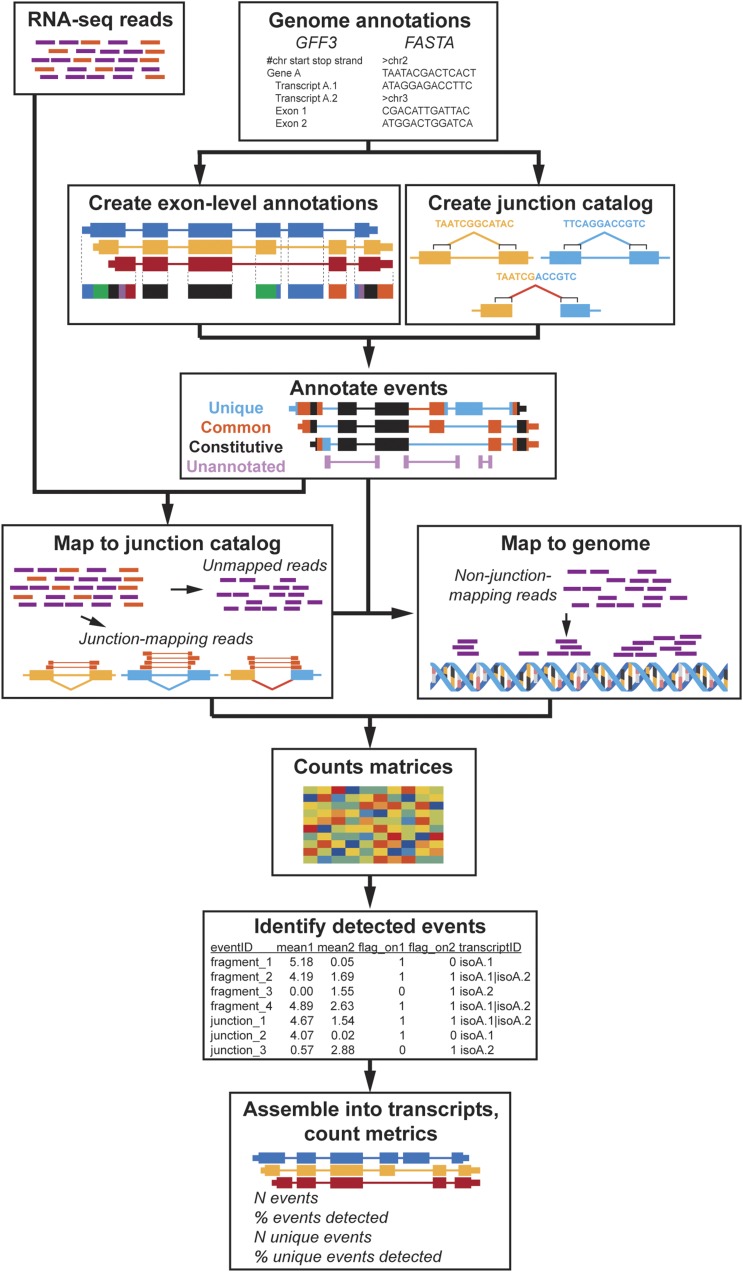
The Event Analysis workflow. Event Analysis consists of three main components: (1) annotation generation; (2) alignment and coverage; (3) event detection and transcript identification. First, annotations for exon-level annotations (exonic regions, exon fragments, introns) and junction annotations are generated. A GFF3 file as input to generate annotations for exonic regions, exon fragments, distinct introns and a catalog of all possible, logical junctions within a gene, and a genome FASTA file is used to extract junction sequences to use for quantification. Second, RNA-seq reads are first aligned to junction sequences and coverage is calculated. Reads that do not align to junctions are then aligned to the genome to calculate the coverage of exonic regions, exon fragments and introns. Third, the output set of counts from alignments are then analyzed to determine what transcriptional events (exonic regions, exon fragment, introns, and junctions) are detected and what transcripts likely present/absent in a given condition.

To apply EA to data, reads are mapped to the junction catalog (Figure 3). For the RNA-seq data used here, duplicate reads are removed and no other processing/trimming is performed. Distinct (non-duplicate) RNA sequence reads are aligned as single-ended reads to the set of cataloged junction sequences using the Bowtie1 algorithm (version 0.12.9) (Langmead *et al.* 2009), allowing for only a single alignment per read (parameter “-m 1”) and for up to three mismatched nucleotides (“-v 3”).The “–tryhard” parameter, and the “best” alignment in terms of stratum were reported using the options “–best–strata–chunkmbs 1024”.

#### Mapping to the genome:

The next step of EA is to map reads not mapped to the junction catalog to the genome. Reads unmapped to junctions were mapped to the genome (GRCm38/mm10 version for the mouse data, GRCh37/hg19 version (release 73) for the T1D data) using BWA-MEM (version 0.7.12) (Li 2013). Reads mapping to exon fragments were identified using the BED file for the genomic regions defining these events. Coverage is summarized as the average number of reads per nucleotide (APN = number of reads aligning to region / region length). For the simulated data, detection was defined as an APN > 0 in at least three of the six samples. For the mouse neural data, detection was defined as an APN > 0 for both replicates. For the T1D data, detection was defined as an APN > 5 in at least 50% of subjects per cell type (Newman *et al.* 2017).

#### Filtering transcripts:

The EA process continues with a summarization of the events as they map to transcripts. Each transcript consists of a series of events (junctions and fragments) (Figure 1). To identify transcripts that may be expressed and filter those that are likely not expressed, EA uses simple measures that capture the elements determining the likelihood a transcript is expressed: coverage, the proportion of events detected, and the proportion of unique events detected. EA outputs a tab-delimited file containing these simple summary statistics. Users can use this to decide what combination of detection, coverage and uniqueness is to be used to filter transcripts. For example, transcripts with no reads mapping to them are clearly not expressed and can be filtered.

For each transcript, coverage is calculated by combining all of the events for that transcript. Since the events are of varying lengths, EA reports the average number of reads per nucleotide (reads in region/ length of the region: APN). APN can be interpreted directly: an APN of 2 means that an average of 2 reads cover that region. The proportion of events detected is calculated by counting the number of these events with coverage (detected; APN > 0) and dividing this by the total number of events in a transcript. Similarly, for transcripts with unique events the proportion of unique events detected is calculated. The inclusion of non-expressed transcripts in the reference can impact the expression estimates of transcripts that are expressed (Liu *et al.* 2016b; Tardaguila *et al.* 2018). Using the measures proposed, it is possible to filter transcripts. For example, a transcript with no unique events but only 10% of its events detected is a candidate for filtering. The measures, coverage, proportion of events detected and proportion of unique events detected are all given in the output of the code provided and so the users can set their own filtering criteria. For the purposes of this manuscript, we focus on several specific references: the complete RefSeq transcriptome (*i.e.*, no filtering), a reduced reference of transcripts with all their associated events detected at APN > 0, and a reduced reference of transcripts with at least 75% of their associated events detected at APN ≥ 5.

### Splice aware mapping: STAR

Reads were aligned to the mm10 genome using STAR (version 2.5.4b) (Dobin *et al.* 2013), using the alignment mode “EndToEnd” (no soft-clipping), minimum allowable read junction overhang was set to 16 bp, a maximum allowable junction overhang of 40 bp, up to three mismatches and no multimapping alignments were allowed. Junctions were converted to genome in the format compatible with AStalavista naming (chromosome, last position of donor exon, first position of acceptor exon, strand) (Foissac and Sammeth 2007).

### iReckon

Genome alignments and junctions identified using STAR (see above) were used as input. Annotations consisting of a 12-column BED file of transcripts (including genomic start positions and lengths of each exon) was used to guide transcript assembly. Alignments to transcript sequences were carried out using BWA-MEM (version 0.7.15) (Li 2013) as per the iReckon user guide, and all default parameters were selected.

### Estimating transcript abundance using RSEM and eXpress

We estimated transcript abundance using RSEM (version 1.2.28) (Li and Dewey 2011) and eXpress (version 1.5.1) (Roberts and Pachter 2013). For RSEM, references were prepared using the tool ‘rsem-prepare-reference’, the set of transcript sequence for a given transcriptome (complete RefSeq, PacBio, reduced references, etc.) and a tab-delimited gene-to-transcript index file as input, as described in the RSEM user guide. The default aligner (Bowtie, version 0.12.9) was used for mapping. For the mouse neural data, reads were aligned single-end, with a mean fragment length of 80 bp and standard deviation of 50 bp. 95% confidence intervals and posterior mean estimates of transcript abundance were also requested. The read start position distribution was estimated from the data. Default settings for RSEM for all other parameters were used. For the T1D data, reads were aligned as paired-end and all other parameters were the same as the mouse neural data.

For eXpress, reads were first aligned to transcript sequences using BWA-MEM version 0.7.15 (Li 2013) with default parameters. Output SAM files were then analyzed using eXpress to estimate abundances of transcripts in each reference transcriptome. Mean fragment length of 80bp and with a standard deviation of 50 bp was set for eXpress, with all other parameters left at their default settings. We estimate transcript abundances for the complete RefSeq transcriptome and two the EA reduced references and the iReckon transcriptome. All iReckon transcripts assembled in each NPC replicate were combined and transcripts present in both samples (based on identifiers and genomic coordinates) were combined into a single transcript (iReckon “union” transcriptome).

### Evaluation of the RNA-seq data

To estimate concordance of transcript estimates between replicates, we first calculated the simple agreement between replicates as the proportion of transcripts detected in both replicates. Transcripts were then binned based on their TPM estimates (transcripts per kilobase million) estimates: “no expression” (TPM = 0 in both replicates), “very low expression” (minimum log-TPM: 0 - 0.5), “low expression” (minimum log-TPM: 0.5 - 2), “moderate expression” (minimum log-TPM: 2 – 4) and “high expression” (minimum log-TPM: 4 or greater). This approximately corresponds to no expression, 25^th^ percentile of expression (“very low expression”), 25^th^ to 75^th^ percentile of expression (“low expression”), 75^th^ to 90^th^ percentile of expression (“moderate expression”), and 90^th^ to 100^th^ percentile of expression (“high expression”). The coefficient of variation (CV) for each TPM bin was then calculated. Replicates are further compared by generating Bland-Altman plots (Bland and Altman 1986).

### Validation of Event Analysis, STAR and iReckon via simulation

The two simulated datasets generated for this study (Simulations 1 and Simulation 2) were used to evaluate and compare the performances of EA and STAR in terms of junction identification, and EA and iReckon in terms of estimated transcriptomes. To compare EA and STAR, junctions were identified and quantified. As STAR only reports the number of reads mapping to a particular junction, the APN for each junction sequence in each sample was estimated by dividing the number of mapped reads reported to the expected junction size (*i.e.*, twice maximum allowable junction overhang). EA cataloged junctions and STAR junctions were matched on genomic coordinates (chromosome, last position of donor exon, first position of acceptor exon, strand). The number of junctions detected (APN > 0) and at different levels of support (APN ≥ 2, 5, 10) by each method, by both methods (intersection of EA and STAR), and by either method (union of EA and STAR) was compared with the number of expected junctions (*e.g.*, the number of annotated junctions from transcripts in the simulation).

The reference reduction using EA was compared with the transcript reassembly of iReckon. For EA, three reduced reference transcriptomes were created: (i) 100% events detected at APN > 0; (ii) at least 75% of events detected at APN > 0; and, (iii) at least 75% of events detected at APN ≥ 5). From the iReckon results, two transcriptomes were used: one consisting of transcripts observed all simulated samples, and one consisting of transcripts observed in at least one simulated sample. Transcripts from these simulations were classified into the following categories: (1) transcripts that were used for read simulation and were correctly identified by EA/iReckon; (2) “related RefSeq transcripts”, which are transcripts not selected for simulating reads but are from the same gene as a transcript used for read simulation; (3) “related non-RefSeq transcripts”, which are transcripts assembled by iReckon but not in the RefSeq annotations, and are from the same gene as a transcript used for read simulation; (4) “unrelated transcripts”, which are those transcripts that are not from genes used to simulate reads; and (5) “missing transcripts”, which are those that were used for read simulation but were not identified by EA/iReckon.

### Validation of Event Analysis With PacBio sequenced transcriptome

The set of transcripts identified using PacBio sequencing (Tardaguila *et al.* 2018) was used for validation. Sequences for each detected exon fragment and junction were compared to the PacBio transcript sequences. Transcripts were compared to PacBio using MegaBLAST (Camacho *et al.* 2009) and events/transcripts were considered validated if the alignment covers at least 90% and had no gaps or mismatches.

### Validation of iReckon-assembled transcripts With PacBio sequenced transcriptomes

To determine how well iReckon reassembles transcripts, the set of transcripts identified using PacBio sequencing (Tardaguila *et al.* 2018) was used for validation. Sequences for each iReckon transcript for each NPC replicate were extracted using BEDtools (version 2.17.0; (Quinlan and Hall 2010)) from the output GTF files and compared to the complete set of 16,104 PacBio transcript sequences using the MegaBLAST algorithm (Camacho *et al.* 2009). BLAST alignments were classified based on the length of the BLAST hit (100% of the iReckon transcript sequence matches a PacBio transcript with more than 10bp different in length between iReckon and PacBio transcripts, at least 90%, 75% or 50% of the iReckon transcript sequence matches a PacBio transcript), and whether there were no mismatches or no more than 5 mismatches nucleotides. iReckon transcripts with gaps or multiple fragmented hits to the same PacBio transcript were excluded from analysis.

For identifying potential RefSeq matches to the iReckon-assembled transcripts, a similar BLAST alignment of iReckon transcripts to the complete RefSeq transcriptome was performed using the MegaBLAST algorithm. We only considered BLAST hits with at least 90% of the sequences matching, allowing for up to five mismatched nucleotides.

### Software availability

The bioinformatics workflow can be found in Figure 3. All code is in python and scripts and documentation can be found at https://github.com/McIntyre-Lab/events.

### Data availability

The mouse neural dataset analyzed in this study is available from the NCBI Sequence Read Archive under accession number SRP101446. The T1D dataset used in this study is available from the dbGaP repository under accession number phs001426.v1.p1. The simulated datasets used to evaluate the performance of EA, iReckon and STAR as well as all code pertaining to the analyses presented in this manuscript can be found at https://github.com/McIntyre-Lab/events. Supplemental material available at Figshare: https://doi.org/10.25387/g3.6205793.

## Results

### Simulation results

#### Junctions:

EA uses mapping to a catalog of junctions for the detection of quantification of junction sequences. An alternative to EA is to map to the genome in a splice aware fashion. We compared the junction detection rate of EA with that of the splice-aware aligner STAR (Dobin *et al.* 2013), using alignment parameters were comparable to those used for mapping to the junction catalog to enable direct comparisons.

In Simulation 1, we found that EA and STAR identify almost the same set of junctions, with EA detecting a few more true junctions than STAR (File S1, Table S1.3). STAR detected ∼100 true junctions that EA did not, and these are almost all complex multi-junction alignments involving microexons (File S1, Table S1.3). Without annotation of microexons (defined as exons of 51 nucleotides or fewer in length) in the reference these events will be missed by any reference-based approach, including EA. However, papers carefully looking at microexons point to the difficulty in capturing these events (Florea *et al.* 1998; Wu *et al.* 2013; Irimia *et al.* 2014; Ustianenko *et al.* 2017).

In Simulation 2, we found that the junctions EA and STAR identify are also largely concordant (File S1, Table S1.5). EA detects 17 annotated junctions that STAR did not. Of the nine unannotated junctions included in this simulation, EA identified all of them, while STAR missed three (File S1, Table S1.6).

#### Transcripts:

For Simulation 1, using the definitions APN > 0 and 100% of the events detected, EA detects 93% of all transcripts simulated and an additional 25,368 transcripts cannot be eliminated from consideration due to similarity with simulated transcripts (File S1, Table S1.4). At APN ≥ 5 combined with the requirement that 75% of events be detected; 90% of the true transcripts are detected 24,201 additional transcripts cannot be eliminated from consideration. iReckon detects fewer (80%) of all simulated transcripts in at least one sample but only 66% in all samples and generates an almost equal number of unrelated transcripts. (File S1, Table S1.4).

For Simulation 2, EA correctly retained more transcripts than iReckon correctly reassembles (File S1, Table S1.7). Using an event detection criteria of APN > 0 and requiring 100% of events detected, EA identified 90% of the 467 simulated RefSeq transcripts, as well one additional, unrelated transcript. When the event detection criteria was set to APN ≥ 5 and transcripts with at least 75% of events detected were retained, EA identified all but one of the 467 RefSeq transcripts and no unrelated transcripts (File S1, Table S1.7). iReckon correctly assembled 391 of the 467 simulated transcripts, and assembled an additional 565 unrelated transcripts (File S1, Table S1.7). When only transcripts in common to all samples were included, iReckon only retained 180 of the 467 transcripts (File S1, Table S1.7).

### Detection of expression in mouse neural progenitors

**Figure 4:**
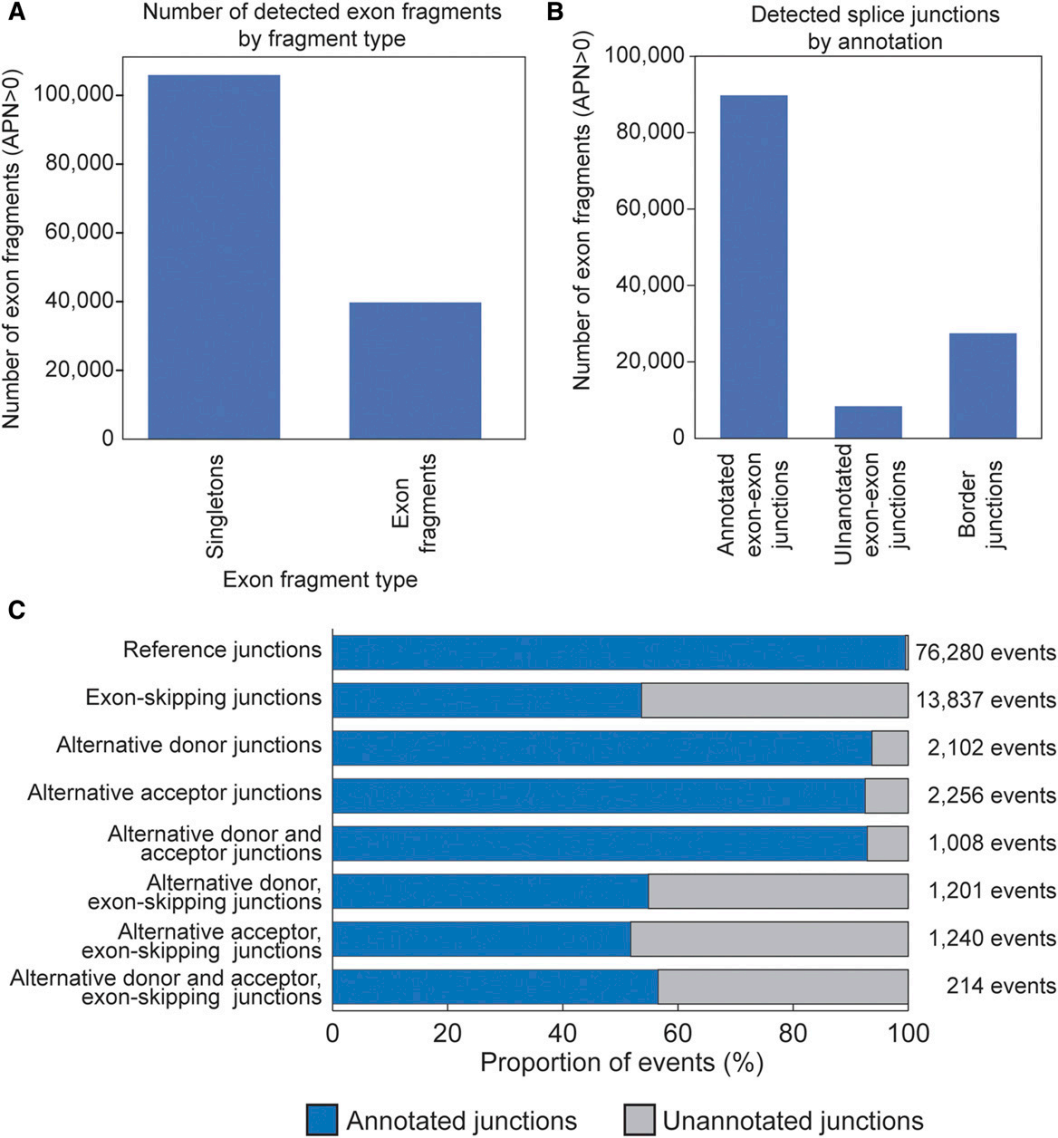
Events detected in cultured mouse NPCs. (A) Exons and exon fragments detected in mouse NPCs. (B) Splicing events by event type detected in mouse NPCs. (C) Detected exon-exon junctions by event annotation. Blue bars indicate exon-exon junctions annotated to known transcripts, gray bars indicate exon-exon junctions that are not annotated to known transcripts.

**Figure 5:**
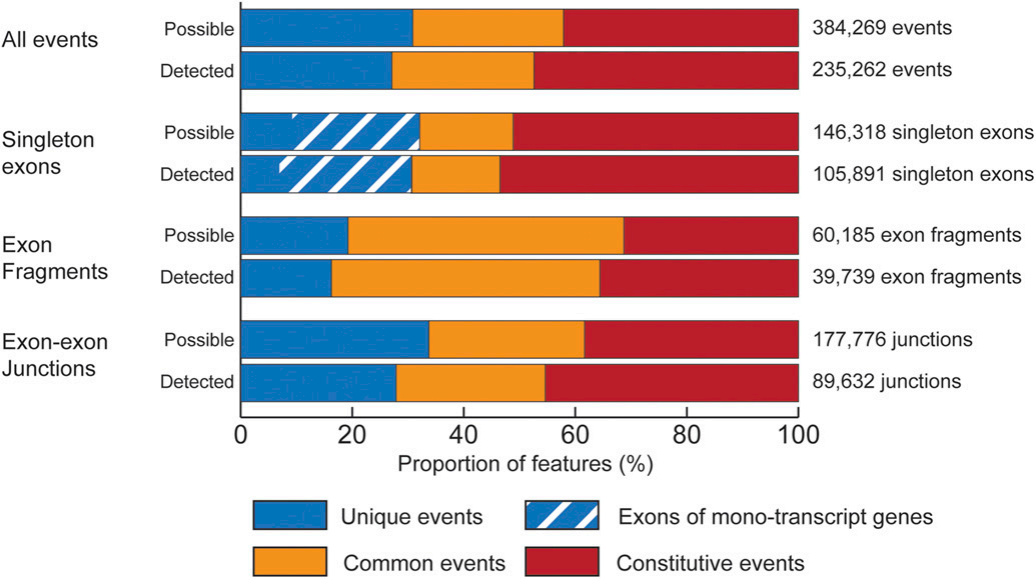
Transcript-specificity of events. Transcriptional events (single-exon exons, exon fragments, junctions) are divided on the basis of transcript-specificity (unique, common, constitutive) Exons of mono-transcript genes are a special case of single-exon exons as these are both unique and constitutive. These are indicated as a subset within unique single-exon exons, and are included within the total set of unique events. The majority of detected events correspond to those constitutively included in all known transcripts for a gene or set of genes.

In cultured mouse NPCs, a total of 105,891 single-exons and 39,739 exon fragments of length at least 10 bp, and 125,716 junctions were detected in 20,875 genes (Figure 4A and 4B). This included 89,812 exon-exon junctions that are annotated to at least one transcript (71% of detected junctions), 8,412 junctions not annotated to any known mouse RefSeq transcript (7% of detected junctions), and 27,492 border junctions (22% of detected junctions; Figure 4B and 4C). The majority of transcript events are constitutively included in all known transcripts for a gene or set of genes (Figure 5).

### Validation of novel events

**Figure 6:**
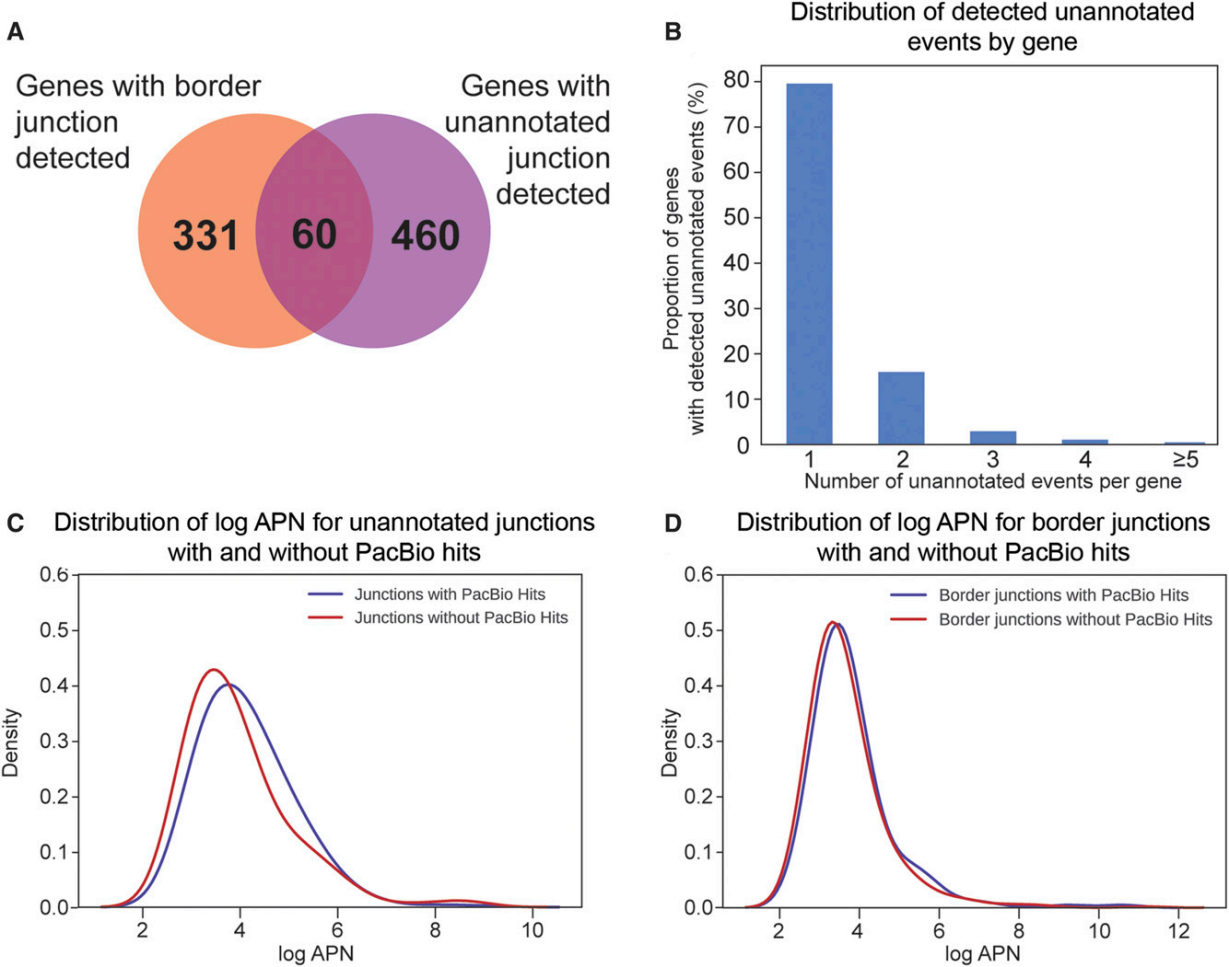
Unannotated splicing events detected in mouse NPCs. (A) Genes with detected unannotated events. (B) Distribution of the number of detected unannotated splicing events per gene. Density plot of the log-APN distribution between detected unannotated junctions (C) and border junctions (D), with and without BLAST hits to the PacBio transcriptome.

The set of detected unannotated splicing events (unannotated junctions and putative IR events) identified in EA are potentially from novel transcripts. We identified 583 putative novel junction events with both flanking exons detected from 520 genes (Figure 6A); most of these genes only have single unannotated junction (Figure 6B). We compared this set of putatively novel junctions against PacBio transcripts to determine the proportion of putative novel events with supporting evidence in the PacBio transcriptome. We found that of 69% were captured in the PacBio transcriptome. Similar to annotated events, novel events captured in PacBio transcripts have a slightly higher mean APN values compared to putatively novel events not present in the PacBio transcriptome (Figure 6C; Figure S1, Additional File 1). The 31% of the unannotated junctions not found in the PacBio data may be from transcripts with lower levels of expression, or they may be false positives.

Border junctions may represent retained introns, novel donors, novel acceptors or incomplete messenger RNA processing. From the set of 154,934 introns, their associated border events and 5′ donor exonic regions, only those with the junction and exonic region detected (APN ≥ 5) were analyzed further (505 border junctions from 391 genes). This set of 505 were classified into their likely sources as described in Figure 2, and the results for confirmation with PacBio are on average 32% (Table 3).

**Table 3:** Border junctions and the overlap with PacBio

Classification	Number of junctions (Junctions with PacBio hit)
Possible IR	123 (52)
Possible novel donor	37 (8)
Ambiguous IR (possible IR and possible novel donor)	11 (6)
Possible unprocessed transcript	334 (94)

### Using Event Analysis as a prior for estimation of transcript quantity

**Figure 7:**
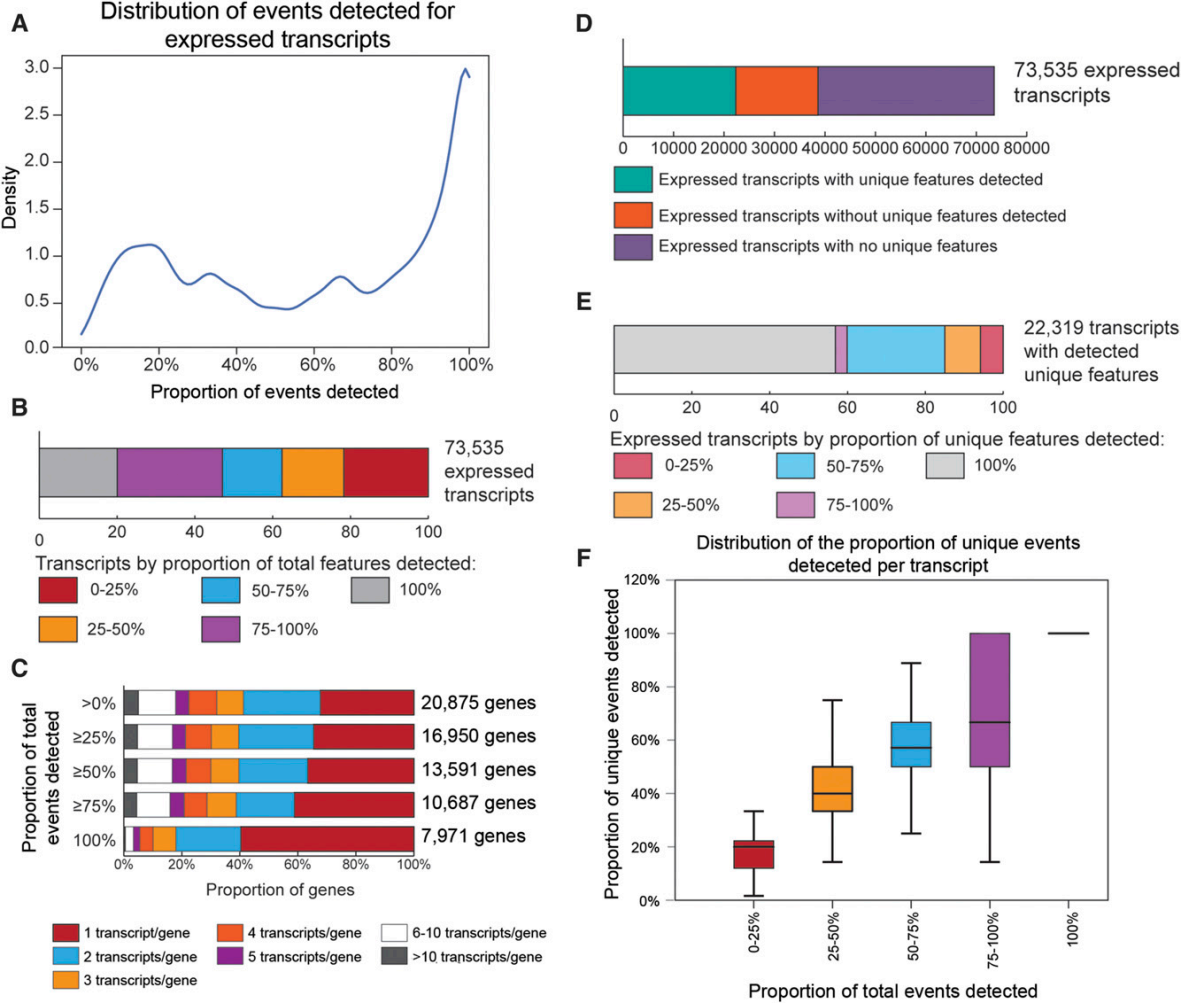
Distribution of the total events detected per transcript. (A) Density plot of the distribution of proportion of total events detected. (B) Proportion of transcripts by the proportion of total events detected. (C) Distribution of number of transcripts detected per gene. (D) The proportions of transcripts with at least one unique event detected, transcripts with unique events but none detected, and transcripts with no assigned unique events. (E) Distribution of the expressed transcripts with at least one unique event detected by the proportion of unique events detected. (F) Scatterplot of number of unique events and proportion of unique events detected per transcript. Most of the transcripts with detected unique events typically had fewer than 3 unique events detected, and about 61% of these 22,319 transcripts had only a single unique event detected.

One of the applications of EA is to filter transcripts not likely expressed (*e.g.*, transcripts with few or none of their associated events detected), and thus define a reduced reference. This reduced transcriptome can then be used in transcript quantification methods. The expectation is that reducing the complexity of a reference transcriptome yields better estimates of transcript abundance (Tardaguila *et al.* 2018). The agreement between replicates was calculated for the RSEM quantification based on the complete RefSeq transcriptome (∼160,000 transcripts in mouse) as a baseline and overall 17% of transcripts disagreed between replicates in terms of detection (Table 4). The CV of lower expressed transcripts was high, particularly for very-lowly expressed transcripts (average CV = 96.290), whereas highly expressed transcripts had a much lower CV (average CV = 19.234; Table 4 and Figure S2, Additional File 1). Concordance at low levels of expression is known to be problematic (McIntyre *et al.* 2011; Tardaguila *et al.* 2018); low expression events (defined reads per kilobase million (RPKM) < 5) typically have a much higher variance (McIntyre *et al.* 2011), and this is observed with the both the variances of very-low and low expression transcripts (RPKM of 5 is approximately equivalent to a log-TPM of 2). There were 20,875 genes with at least one exonic region detected corresponding to 75,926 annotated transcripts. 2,391 transcripts from the set of expressed genes had none of their exons or junctions detected and were removed for a total of 73,535 possible expressed transcripts (Figure 7). The agreement among replicates based on this transcriptome was similar to that of the complete RefSeq transcriptome (Table 4), and the average CV only marginally improved for the lower expressed transcripts (Supplementary Figures 2 and 3, Additional File 1), indicating that RSEM already effectively removes clearly unexpressed transcripts and there is no incremental benefit to only removing these transcripts.

**Table 4:** Agreement statistics of detection of transcripts per transcriptome

Transcriptome	Event detection	Proportion events detected	Total transcripts (N)	Not detected (N)	Detected in NPC replicate 1 (N)	Detected in NPC replicate 2 (N)	Detected in both (N)	Disagreement (%)
RefSeq	All	n/a	128,631	62,940	10,441	11,528	43,722	17.08%
EA	All	>0%	73,535	28,071	6,894	7,621	30,949	19.74%
EA	APN > 0	≥50%	45,883	12,375	4,443	3,306	25,759	16.89%
EA	APN > 0	≥75%	34,622	8,006	3,078	2,180	21,358	15.19%
EA	APN > 0	100%	14,734	970	563	532	12,669	7.43%
EA	APN ≥ 5	≥50%	20,336	3,871	1,487	918	14,060	11.83%
EA	APN ≥ 5	≥75%	13,740	1,868	757	439	10,676	8.70%
EA	APN ≥ 5	100%	3,815	103	37	17	3,658	1.42%
PacBio	All	n/a	16,104	1,002	508	303	14,291	5.04%
PacBio	APN > 0	>0%	6,286	175	61	41	6,009	1.62%
PacBio	APN ≥ 5	>0%	6,078	168	56	29	5,825	1.40%

The proportion of total events, and the proportion of unique events that were detected was calculated for each RefSeq transcript. The transcripts fell into two distinct modes, those with at least half of their associated events detected and those with a much lower proportion detected, with approximately 20% of the potential transcripts with less than 25% of their events detected (Figure 7A and 7B). The number of transcripts per gene was lower when requiring all events to be detected (Figure 7C). There were 34,900 expressed transcripts without any assigned unique events (*i.e.*, non-resolvable transcripts (Figure 7D and 7E). Because these transcripts are not resolvable, their detection is by definition ambiguous. Among the transcripts with detected unique events, there was little evidence that the number of unique events assigned to a transcript affected the proportion that were detected per transcript, but that the proportion of unique events detected was a function of the amount of total events detected (Figure 7F).

We reasoned that transcripts consistently detected across their length were more likely to be present than those inconsistently detected. We then examined the agreement between replicates in transcript detection and variation in transcript estimates after eliminating transcripts with different proportions of their events detected and at different levels of average coverage. There was a distinct improvement in replicate agreement and estimate precision when limiting the set of possible transcripts to those with more consistent within transcript detection (Table 4; Supplementary Figures 2 and 3, Additional File 1). Restricting the list of possible transcripts before quantification to those for which there were at least 5 reads on average for 75% or more of their events resulted in an average CV of 36.051 for lowly expressed transcripts, and an agreement of 91% between replicates for detection of transcripts. Restricting the list of possible transcripts before quantification to those for which there were at least 5 reads on average with 100% of their events detected resulted in an average CV of 28.249 for lowly expressed transcripts, and an agreement of almost 99% between replicates for detection of transcripts.

### Validation of Event Analysis transcriptomes With the PacBio transcriptome

**Figure 8:**
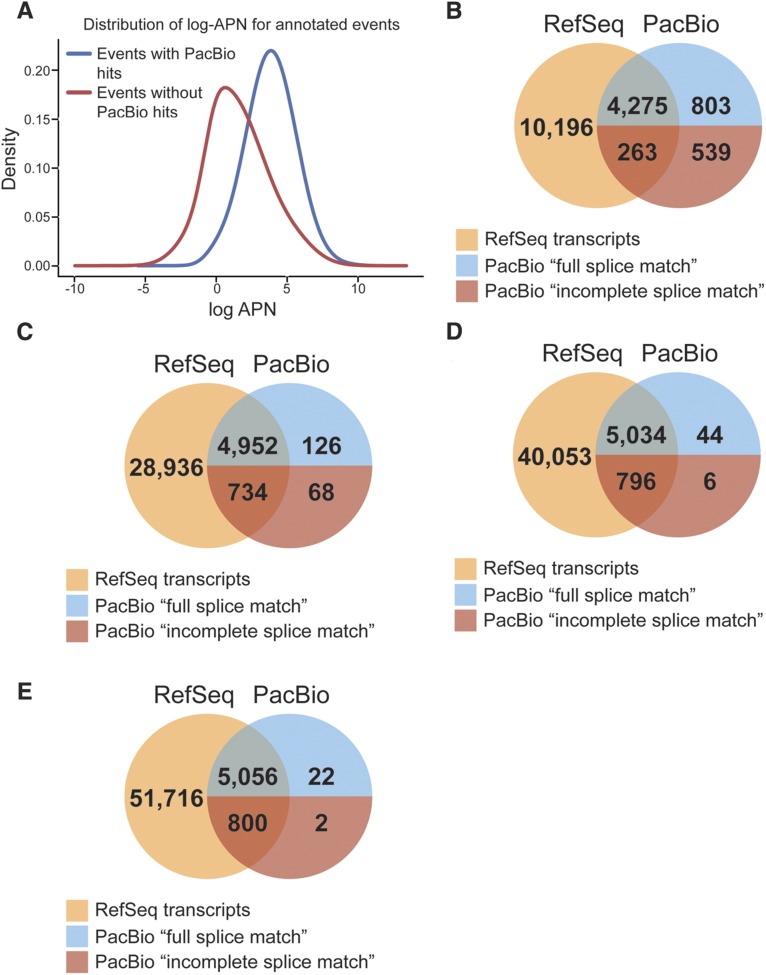
Comparison of Event Analysis with PacBio sequenced transcripts. (E) Distribution of log-APN of annotated events with and without PacBio hits. Blue line represents the of log-APN distribution of annotated events with PacBio hits, red line represents the of log-APN distribution of annotated events with no PacBio hits. Overlap between the Event Analysis trancriptome (yellow) against the PacBio transcriptome (blue = complete splice match; red = incomplete splice match), for EA transcripts with 100% of events detected (B), at least 75% of events detected (C), at least 50% of events detected (D), and at least 25% of events detected (E).

From the set of 20,785 genes not filtered from the RNA-seq data, we examined the how many of the detected annotated events – 39,739 exon fragments, 105,891 single-exons and 89,632 annotated junctions – were present in the set of PacBio transcripts. PacBio transcripts were assigned reference transcript identifiers (RefSeq or Ensembl) if: (1) the PacBio transcript matched at all splice junctions of a reference transcript (“full splice match” or FSM (Tardaguila *et al.* 2018)), or (2) splice junctions of the PacBio transcript matched consecutive but not all splice junctions of a reference transcript (“incomplete splice match”; ISM (Tardaguila *et al.* 2018)). Monoexonic PacBio transcripts were considered a “full splice match” if they corresponded to a monoexonic reference transcript, or “incomplete splice match” if they were corresponded to multiexonic reference. Of the total 235,262 events detected, 66,646 events from 4,426 genes (3,796 fragments, 19,388 single-exons and 43,462 junctions) matched at least one PacBio transcript (28% of events, 21% genes). We hypothesize that the remaining RNA-seq mapped reads to annotated events are representative of transcripts that are not captured by PacBio due to lack of sequence depth, and thus will have lower short-read coverage than annotated events that mapped to PacBio transcripts. To test this we compared the APN in events captured by PacBio to events not captured by PacBio. (Figure 8A).

Of the ∼16,000 PacBio transcripts (Tardaguila *et al.* 2018), there are 9,653 that match to RefSeq transcripts, and of these 5,880 have all events unambiguously annotated to a single gene. Only 24 of the PacBio transcripts (<1%) had fewer than 25% of their total associated events detected. Of the remaining set of 5,856 PacBio transcripts, all were included in the set of probable transcripts identified by EA (Figure 8). EA transcripts were categorized based on whether they had unique events, the proportion of total events detected, and the average coverage. The number of transcripts in each category for the 73,535 EA transcripts and 5,856 PacBio Transcripts are listed in Table 5. Eliminating EA transcripts with fewer than 75% of their annotated events detected (38,913 transcripts with <75% of total features detected, APN > 0) reduces the complexity of the EA transcriptome by 53% (35,342 transcripts remaining) while missing only 3% of the PacBio transcriptome (194 of 5,880 PacBio transcripts). If a higher APN threshold is used to determine event detected (*e.g.*, APN ≥ 5) with a lower frequency of event detection (50%) results in less than 1% of the true PacBio transcriptome is missed and the EA transcriptome is much less complex (20,336 transcripts; Table 5). A similar number of transcripts, with a similar set of PacBio transcripts are identified with two thresholds (100% events detected (APN > 0), 14,734 transcripts; ≥75% events detected (APN ≥ 5), 13,740 transcripts). These two sets have 9,310 transcripts are in common between the two possible transcriptomes. This overlap captures 79% of the PacBio transcripts that match to either of these possible transcriptomes. This demonstrates that the choice of parameters impacts the proportion of PacBio-validated transcripts that can be captured in the possible, reduced transcriptomes and that selecting a single set of parameters will included more PacBio transcripts than the intersection of two parameter sets.

**Table 5:** Distribution of transcripts by proportion of events detected Number in parenthesis is the number of transcripts with a matching PacBio transcript

Proportion of events detected (%)	APN > 0 event detection level		APN ≥ 5 event detection level	
	Transcripts with no unique events	Transcripts with unique events	Transcripts with no unique events	Transcripts with unique events
**1–24%**	9,342 (4)	6,621 (20)	3,248 (80)	4,147 (195)
**25–49%**	6,628 (11)	5,061 (15)	2,185 (82)	3,193 (243)
**50–74%**	5,837 (26)	5,424 (118)	2,544 (206)	4,052 (465)
**75–99%**	8,809 (408)	11,799 (740)	3,499 (851)	6,426 (1,505)
**100.00%**	5,004 (1,307)	9,730 (3,231)	1,053 (480)	2,762 (1,566)
**Total**	34,900 (1,756)	38,635 (4,124)	12,529 (1,699)	20,400 (3,974)

### Event Analysis improves transcript abundance estimates

The mouse neural data used thus far demonstrates that using a reduced transcriptome as a prior improves the concordance between technical replicates and also captures most of the same reference transcripts as PacBio. An RNA-seq dataset of 3 lymphocyte populations (CD4^+^, CD8^+^ and CD19^+^ lymphocytes) from 81 T1D cases was used to determine whether a reduced transcriptome also reduces the variability of transcript estimates in a larger set of RNA-seq data.

In T1D data, a total of 16,156 single-exons and 67,888 exon fragments of length at least 10 bp, and 38,367 junctions were detected from 8,700 genes in at least one cell type. This included 34,421 exon-exon junctions that are annotated to at least one transcript (90% of detected junctions), 402 junctions not annotated to any known human AceView transcript (1% of detected junctions), and 3,544 border junctions (9% of detected junctions). We used the criteria APN ≥ 5 and 75% of more events detected to a filter the reference with a resulting reduced transcriptome consisting of 26,437 transcripts (Figure S4, Additional File 1).

**Figure 9:**
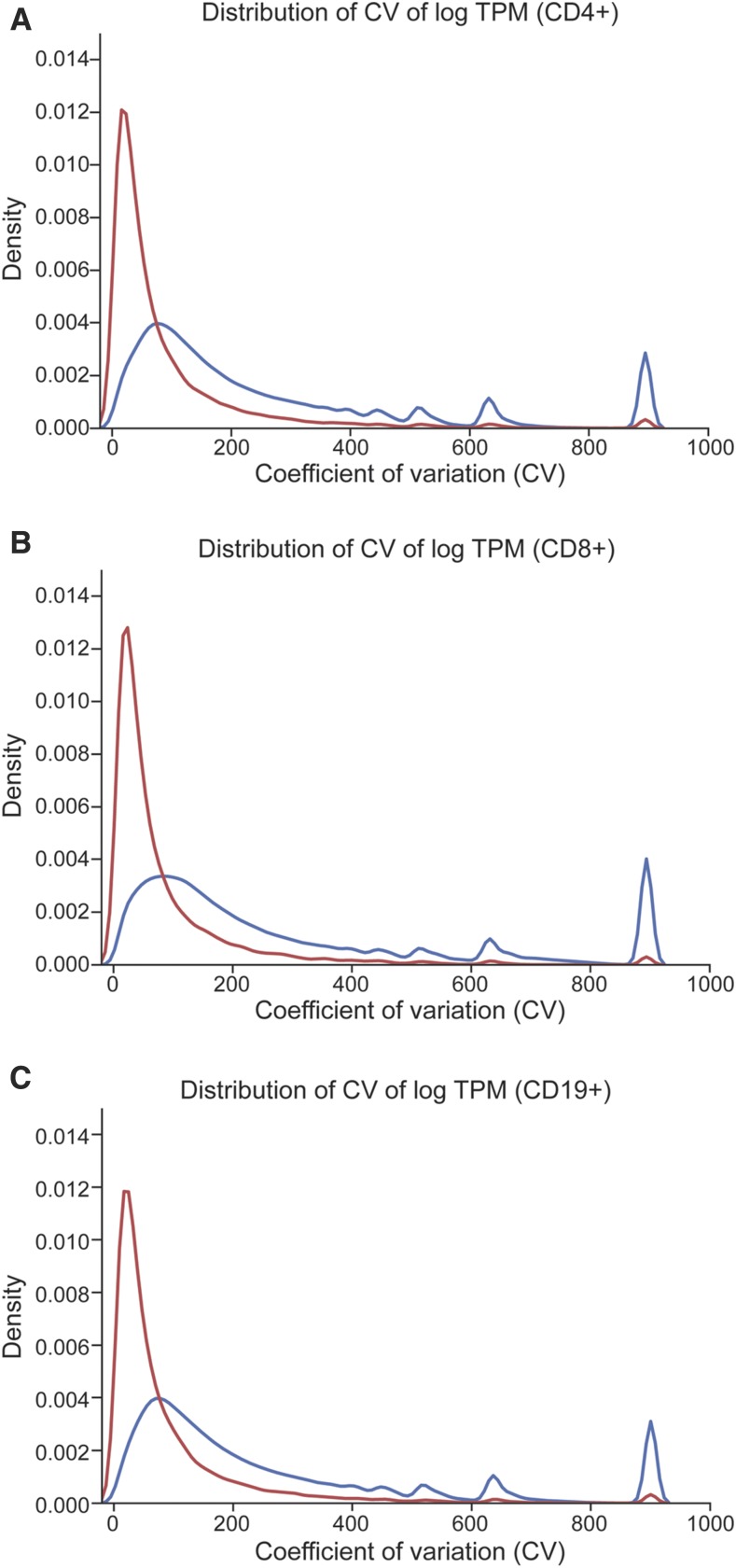
Distribution of the coefficient of variation of transcript estimates in T1D lymphocyte data. The distribution of CV between samples from the quantification for all AceView transcripts (blue line) and the reduced set of transcripts (red line) for (A) CD4^+^, (B) CD8^+^ and (C) CD19^+^ lymphocytes.

We then examined whether using this reduced transcriptome reduced variability in abundance estimates compared to the complete transcriptome (∼596,000 AceView transcripts in human). As there are 81 subjects who have been examined for 3 lymphocyte cell types, we calculated the coefficient of variation (CV) for each transcript/cell type after quantitation using RSEM for the complete AceView transcriptome (Figure 9, blue line) and for the reduced reference (26,437 transcripts). The distribution of the CV across transcripts was compared between the full reference and the filtered reference (Figure 9). For each transcript the CV between the two was compared and if the CV was larger in the full reference it was scored (+) and of the CV was smaller it was scored (-). The null hypothesis that the distribution is random was tested using a sign test (*P* < 0.0001). The overall distribution showed a dramatic decrease in the CV in the reduced reference and per transcript the CV was lower in the filtered reference. Together this indicates a dramatic increase in the concordance of estimates among samples. Similar results were obtained with eXpress (File S2).

As an example, for the T1D data, in the *IKZF3* gene, a lymphoid transcription factor and T1D candidate gene (Morgan *et al.* 1997; Hosokawa *et al.* 1999; Barrett *et al.* 2009), EA reduces the number of transcripts from 18 to four, and quantitation demonstrates there is a shift in expression such that the isoform IKZF3.iAug10 is expressed more frequently in CD4+ and CD8+ T cells than in CD19+ B cells (Supplementary Figures 5 and 6, Additional File 1). This isoform corresponds to the isoform variant 5 of *IKZF3*, which lacks two exons and several zinc finger binding domains (Morgan *et al.* 1997; Hosokawa *et al.* 1999). This enables us to follow up on the most-likely, relevant *IKZF3* transcripts in these cell types without the burden of transcripts that provide little additional information about the transcription of this gene.

### Comparison of mouse NPC data With STAR

STAR identifies ∼24% of the junctions (883 out of 3,691) not present in the current annotation, and ∼95% of the junctions present in the annotation and verified by PacBio reads while EA detects 100% of the junctions present in the annotations. When adding the criteria of at least two reads to support the identification of a junction, STAR identifies only 3% of junctions while EA identifies 76%. STAR identifies 43% (229 out of 536) of the junctions that are novel combinations of existing donor/acceptors and EA detects ∼60% (321/536) of these junctions. Thus, mapping directly to the junction catalog that includes all possible logical junctions improves support of annotated junctions (Tables S3.2 to S3.5, File S3).

### Comparison of mouse NPC data With iReckon

We also compared the set of possible transcripts identified by EA and the transcripts assembled by iReckon against the 5,880 PacBio transcripts with RefSeq identifiers. iReckon assembles 10% of the validation set of transcripts. EA is able to identify as many as 97% of the 5,880 PacBio transcripts, when considering transcripts with at least 75% of their associated events detected (Figure 8; File S4). Even when only transcripts with 100% of their events detected are considered, 77% of the PacBio transcriptome is identified with EA, a substantial improvement over that of iReckon. In addition, the majority of iReckon transcripts are classified as novel transcripts (File S4), and only approximately 13% of the iReckon transcripts have good sequence similarity to the PacBio transcriptome. EA is also unable to eliminate ∼10,000-30,000 additional RefSeq transcripts, owing to the high degree of sequence similarity and event sharing with other transcripts within in the same genes.

## Discussion

At the core of EA is the preparation of a catalog of junctions. Some analysis approaches (*e.g.*, (Katz *et al.* 2010)) restrict the definition of junctions to those present in existing annotations. Others attempt to estimate junctions *de novo* from short reads (Trapnell *et al.* 2009; Dobin *et al.* 2013; Kim *et al.* 2013a). The literature indicates that the vast majority of novel isoforms are assembled from currently annotated exons (Booms *et al.* 1999; Hide *et al.* 2001; Zhou *et al.* 2010; Lim *et al.* 2011; Eswaran *et al.* 2013; Kim *et al.* 2013b; Gabreski *et al.* 2016; Nellore *et al.* 2016; Tardaguila *et al.* 2018). By designing a junction catalog that includes junctions created from all possible logical combinations of existing exons, and mapping to that catalog more true novel junctions are mapped than by mapping to the genome with a splice aware aligner. While completely novel junctions (*i.e.*, junctions with donor and/or acceptor sites not present in the reference annotation) will be missed, we find that in the study here of mouse NPC cells novel junctions and transcripts identified by PacBio are not frequently identified by *de novo* tools STAR or iReckon (Supplementary Files 3 and 4).

EA views each transcript as an annotated set of discrete events. The detection and coverage of each event can be used directly in an analysis or to filter transcripts. Code for this is provided. EA does not rely on complex probabilistic models of transcription nor specific sequencing technology. Using this approach, in mouse neural progenitor cell RNA-seq data EA identified the overwhelming majority of annotated PacBio transcripts (5,686 of 5,880 transcripts, 97%) using the criteria 75% or more events detected (APN > 0). A more stringent definition (least 75% of their events detected at APN ≥ 5) identifies 4,402 annotated PacBio transcripts.

There is a limit to the ability of any RNA-seq based approach to identify transcripts. As EA relies on existing genome annotations, it is not able to identify completely novel exons or splice sites. This is a common limitation for annotation-based quantification approaches. EA does not *de novo* assemble transcripts, as accurate transcript reassembly and resolution from short-read data are known to result in a higher number of false transcripts (Steijger *et al.* 2013; Angelini *et al.* 2014; Bernard *et al.* 2015; Hayer *et al.* 2015; Liu *et al.* 2016a; Song *et al.* 2016) and shown here. Long reads are able to clearly identify novel isoforms, provided adequate quality control mechanisms are applied (Tardaguila *et al.* 2018). Another limitation is the resolvability of individual transcripts. Transcript resolvability is not a limitation of EA but of the uniqueness of transcripts. When we examined presence/absence of unique events among the PacBio transcripts we found that 30% of the PacBio transcripts have no unique events distinguishing them, meaning that these will never be identifiable from short read data alone. Issues with transcript quantification from short read RNA-seq data alone have been acknowledged and discussed (Angelini *et al.* 2014; Kanitz *et al.* 2015; Ding *et al.* 2017; Williams *et al.* 2017; Tardaguila *et al.* 2018).

The newly released pRSEM cleverly tackles the issue of transcript resolvability by leveraging ChIP-seq data. In addition, pRSEM uses a binomial distribution to divide transcripts into two groups, probably expressed and unexpressed, before quantification. Dramatic improvements in transcript quantification were demonstrated using information from ChIP-seq and PolII to estimate the initial transcript set (Liu *et al.* 2016b). Additional methods aim to reduce the number of transcripts to quantify by leveraging long-read sequencing like PacBio to serve as a reference set of transcripts (Au *et al.* 2013; Ning *et al.* 2017), which improve the accuracy of genes model (Tardaguila *et al.* 2018) but at the cost of excluding other, likely-expressed transcripts. However, much like pRSEM, such approaches rely on additional data to determine the likely transcriptome. Other methods that require only short read data to identify reduced reference set have also been successful (*e.g.*, (Mezlini *et al.* 2013; Soneson *et al.* 2016)). One of these, iReckon, which leverages existing annotations to guide the reassembly of transcripts from short reads and quantification of assembled sequences (Mezlini *et al.* 2013). iReckon also creates novel junctions and exons based on read mapping. The overall approach is to create all possible transcripts from the data, then eliminate those with low/no coverage. However, we find iReckon tends to reconstruct many novel sample-specific transcripts not supported by long read data and also reported in the literature (Angelini *et al.* 2014).

Another approach, taken by Soneson *et al.* (Soneson *et al.* 2016), has the transcript abundance algorithm guide what transcripts are likely present. First, all possible transcripts are quantified and the proportion that each transcripts contributes toward the overall expression of it corresponding gene. Transcripts with low abundance and that contribute little toward to its gene’s expression are removed, and the remaining transcript sequences are re-quantified. While this approach is similar to EA (*i.e.*, elimination of unlikely transcripts based on coverage), it may eliminate transcripts that are actually present but whose reads have been initially misassigned to other transcripts. For example, from the mouse neural data used in this manuscript, elimination of the transcript NM_025668 as suggested by this algorithm also eliminates the only PacBio-sequenced transcript for the *Spcs2* gene, and assigns all reads to a different transcript, XM_006508117 (Tardaguila *et al.* 2018). This read misassignment is likely due to variability of the length of the 3′ UTR: while the junctions of the PacBio transcript completely match those NM_025668, the 3′UTR of the PacBio transcript better matches that of XM_006508117 (Tardaguila *et al.* 2018). This occurs because RSEM infers the likely transcript based on coverage in the 3′ UTR. This also highlights the need for a good-quality reference transcriptome that best reflects the actual expression of the experimental system in use.

Providing a reduced set of reference transcript sequences based on the expression of individual transcript events dramatically improves the performance of both RSEM (Li and Dewey 2011) and eXpress (Roberts and Pachter 2013). The agreement between replicates jumps from 80 to above 90% and is as high as 99%, with a dramatic reduction in estimates of variability. The advantages to EA are: (1) only RNA-seq data are required; (2) no model for transcript generation is needed; (3) no model for bias in sequencing technology is needed; and (4) the degree of over/underspecification of the likely transcriptome can be easily adjusted based on the goals of the particular study by applying different thresholds (see Figure S4, Additional File 1). The improved transcript estimates, in turn, will improve the accuracy of differential expression analyses.

Junctions and exonic regions can be used directly to test for differential expression. In a comparison of cell types among type 1 diabetes patients, we found that 20% of splicing events were differentially detected, 34% of genes were alternatively spliced among cell types (Newman *et al.* 2017) and the genes implicated in immune function and autoimmunity are enriched for splicing differences (Newman *et al.* 2017).

## Conclusions

EA is straightforward to apply. There are only two relatively simple new steps that are needed- the creation of an expanded junction catalog, and the scoring of transcripts based on simple measures of detection and abundance. Here we demonstrate that the junction catalog successfully identifies novel junctions and that using transcriptional events to reduce the complexity of a probable transcriptome dramatically improves transcript estimates from RNA-seq data. EA provides rich annotation for events (junctions and exons) enhancing our understanding of the effect of splicing in human disease (Akin *et al.* 2016; Newman *et al.* 2017). Examining individual junctions, exons and exon fragments reveals detail in differential detection/expression patterns previously obscured.
